# Identification of bacterial and fungal components in tobacco and tobacco smoke

**DOI:** 10.1186/1617-9625-4-4

**Published:** 2008-07-31

**Authors:** Lennart Larsson, Bogumila Szponar, Beston Ridha, Christina Pehrson, Jacek Dutkiewicz, Ewa Krysińska-Traczyk, Jolanta Sitkowska

**Affiliations:** 1 Department of Laboratory Medicine, Lund University, Lund, Sweden; 2 Institute of Immunology and Experimental Therapy, Polish Academy of Sciences, Wroclaw, Poland; 3 Department of Occupational Biohazards, Institute of Agricultural Medicine, Lublin, Poland

## Abstract

The microbiological composition of tobacco products was studied using culture and chemical analysis (of tobacco leaves) or chemical analysis only (tobacco and tobacco smoke). The chemical analyses utilized gas chromatography-tandem mass spectrometry for determining 3-hydroxy fatty acids, muramic acid, and ergosterol as markers of respectively lipopolysaccharide (LPS), peptidoglycan, and fungal biomass. Mesophilic bacteria dominated in both fresh and cured tobacco leaves; a range of additional bacteria and fungi were also found albeit in minor amounts. The peptidoglycan and LPS concentrations were approximately the same in tobacco leaves as in cigarette tobacco. The concentrations of the measured microbial components were much lower in some cigarettes locally produced in China, Korea, and Vietnam than in cigarettes of international brands purchased in the same countries, and the concentrations in the smoke were in general agreement with the concentrations in cigarette tobacco. No differences in microbial load in tobacco of "light" and "full flavor" cigarettes were seen. Storing cigarettes at high humidity resulted in elevated levels of fungi in the cigarette tobacco leading to increased ergosterol concentrations in the smoke. The fact that tobacco smoke is a bioaerosol may help to explain the high prevalence of respiratory disorders among smokers and non-smokers exposed to second hand smoke since the same symptoms are also commonly associated with exposure to bioaerosols.

## Introduction

Many hundreds of compounds known to contribute to disease development have been identified in tobacco smoke. Both active and second hand smoking causes cancer and a multitude of other diseases such as for example chronic bronchitis and asthma. Three studies [1-3] have revealed that tobacco smoke contains endotoxin (lipopolysaccharide, LPS), a family of inflammatory toxins from Gram-negative bacteria known to cause respiratory disease upon inhalation [4]. Hasday et al. [1] found that the amounts of endotoxin in tobacco were comparable with those of some other agricultural products. While Hasday et al. [1] used a *Limulus *method for measuring endotoxin, Larsson et al. [2] introduced gas chromatography-tandem mass spectrometry (GC-MSMS) for demonstrating 3-hydroxytetradecanoic acid, a unique LPS constituent [5], in cigarette tobacco and smoke. GC-MSMS is a very specific analysis method for unequivocal identification of LPS. Sebastian et al. [3], using the same GC-MSMS method, demonstrated a linear relationship between the number of cigarettes smoked over a 5-h period indoors and air concentrations of endotoxin. Whether bioactive microbial compounds other than LPS, such as for example peptidoglycan and various fungal components, are present in cigarette smoke is not known from the literature.

An integrated method for characterizing microbial composition in environmental samples by GC-MSMS has been developed at our laboratory [6]. The method includes a protocol for preparation and analyzing samples for LPS markers 3-hydroxy fatty acids (3-OH FAs) of 10 – 18 carbon chain lengths, peptidoglycan marker muramic acid (MuAc), and fungal biomass marker ergosterol (Erg). In the present study a modified version [7] of this method was used for 1) measuring 3-OH FAs, MuAc, and Erg in tobacco from cigarettes of international as well as local brands purchased in different countries in Europe and Asia including "light" and "full flavor" cigarettes; 2) analysing the microbiological composition of tobacco leaves during different stages of curing by using both cultivation and determination of 3-OH FAs, MuAc, and Erg; 3) comparing the concentrations of the mentioned microbial markers in cigarette tobacco and smoke.

## Materials and methods

### Tobacco and smoke

Tobacco of cigarettes from altogether 37 packs purchased in different cities in Europe and Asia were studied including i) "light" (1 pack/city) and "full flavor" (1 pack/city) cigarettes of a well-known international brand purchased in Wroclaw, Lund, Shanghai, Seoul, and Hanoi, ii) cigarettes of other different international brands (Wroclaw 3, Lund 4, Shanghai 1, and Seoul 2 packs, respectively) and iii) popular cigarettes of local origin (Wroclaw 3, St. Petersburg 3, Shanghai 3, Seoul 3, and Hanoi 5 packs, respectively).

Tobacco of "full flavor" cigarettes of the same international brand was analysed after the cigarettes had been stored at 54%, 75%, and 94% relative humidity (RH), each at 20°C and 30°C during 2, 8, and 21 days. Saturated solutions of respectively Mg(NO_3_)_2_, NaCl, and KNO_3 _were used for achieving the desired RH [8]; the cigarettes were stored in closed 1-L glass vials without any direct contact with the respective salt solution.

Tobacco and smoke of cigarettes were analysed after the tobacco had been enriched with bacteria and fungi. In brief, tobacco was removed from 12 "full flavor" cigarettes of an international brand. A suspension (5 ml) of *E. coli *(ATCC 25922) that had been cultivated for 3 days at 37°C on trypticase soya agar (TSA) was added to 6 of the tobacco portions; sterile water (5 ml) was added to remaining 6 portions (controls). The tobacco was dried at room temperature for 72 h. Then, 6 of the tobacco portions (3 bacteria-enriched portions and 3 controls) were analysed for 3-OH FAs and MuAc whereas the remaining 6 portions were used to prepare new cigarettes, the smoke of which was analysed for 3-OH FAs and MuAc (thus, 3 smoke samples from bacteria-enriched tobacco and 3 from the controls). In addition, tobacco and smoke from cigarettes that had been stored for 8 and 21 days at 94% RH, the latter cigarettes visibly colonized by molds (see below), were analysed for Erg. The methods used for generating the smoke and collecting the mainstream smoke particles on Teflon filters have been described elsewhere [2].

### Tobacco leaves

Fresh (F-1) tobacco leaves (light Burley) were collected from a plantation in southern Poland. Leaves from the same plantation were also taken after different time periods of air drying outdoors: for 2 wk (F-2), 6 wk (F-3), and > 6 wk (F-4) *viz*. just before the leaves were shipped to the tobacco manufacturing plant. In addition, samples of air-dried light Burley (B), fire-dried light Virginia (V), air-dried and smoked dark Kentucky (K), and air-dried dark Skroniowski Mocny (SM) tobacco material were obtained from a manufacturing plant in eastern Poland in the forms of air-dried leaves (2007 harvest; stage 1), after curing (2007 harvest; stage 2), and after curing and storage > 1 year (2006 harvest; stage 3).

Cultivation was performed according to Skórska et al. [9]. In brief, chopped plant material (1 g) was suspended in 100 ml of saline with 0.05% Tween 80, and after shaking, serial 10-fold dilutions were made. 0.1-ml aliquots of each dilution were spread on duplicate sets of different agar media. Blood agar was used for cultivating mesophilic Gram-negative and Gram-positive bacteria, eosin methylene blue (EMB) agar (Merck, Darmstadt, Germany) for cultivating Gram-negative bacteria, half-strength TSA (Sigma, St. Louis, MO, USA) for cultivating thermophilic actinomycetes, and malt agar (Difco, Detroit, MI, USA) for cultivating fungi. The blood agar plates and EMB agar plates were incubated for 1 day at 37°C, then 3 days at 22°C, and finally 3 days at 4°C. The malt agar plates were incubated for 4 days at 30°C and 4 days at 22°C. The prolonged incubation at lower temperatures aimed to isolate as wide a spectrum of bacteria and fungi as possible. The TSA plates were incubated for 5 days at 55°C. Colonies were counted and differentiated and the data reported as CFU/g tobacco. The bacteria were identified by using the API 20E, NE (bioMérieux, Marcy l'Etoile, France), and BIOLOG (Biolog, Inc., Hayward, CA, USA) systems. The fungi were identified by using microscopy [9].

### Chemical analysis

Cigarette tobacco, tobacco leaves, and smoke particles (collected on Teflon filters) were dried, weighed, and subjected to hydrolysis. The entire hydrolysates of the particle-containing Teflon filters, a 1/50th fraction of each 200–300 mg tobacco leave hydrolysate sample, and a 1/100th fraction of each cigarette hydrolysate sample were further prepared for GC-MSMS analysis of 3-OH FAs, MuAc, and Erg as described previously [6,7]. Numbers of moles of LPS were calculated by summarizing the number of moles of the 3-OH FAs and dividing by 4 [6].

### Statistical analysis

Independent Student's t-test (STATISTICA 7.1 software, StatSoft, USA) was used; p value < 0.05 was considered as significant.

## Results

### Tobacco

LPS (5.7 – 21.0 pmol/mg), MuAc (1.4 – 10.7 ng/mg), and Erg (0.3 – 12.8 ng/mg) were detected in the tobacco of all of the studied cigarettes. Cigarettes of local brands purchased in China contained significantly less of MuAc and Erg than did cigarettes of international brands purchased in the same stores. Analogously, local cigarettes purchased in Korea contained less Erg than international brands, and local cigarettes purchased in Vietnam contained less LPS and Erg than international brands (Table 1).

**Table 1:** Microbial components in tobacco of cigarettes purchased in different countries.

		**International brands**		**Local brands**		**p-value** ** Int-Loc**
			
		min value	max value	min value	max value	
Sweden		n = 6		n = 0		
	LPS	11.7	21.0	ns	ns	
	MuAc	3.8	8.5	ns	ns	
	Erg	5.3	12.8	ns	ns	

Poland		n = 5		n = 3		
	LPS	7.6	19.1	10.8	15.0	
	MuAc	2.9	9.0	2.5	6.5	
	Erg	2.0	10.2	6.2	9.9	

Russia		n = 0		n = 3		
	LPS	ns	Ns	9.3	12.8	
	MuAc	ns	Ns	2.7	9.4	
	Erg	ns	Ns	6.0	7.9	

China		n = 3		n = 3		
	LPS	7.0	15.2	7.2	8.2	
	MuAc	7.0	7.4	2.0	2.3	*0.000005*
	Erg	7.1	9.1	0.3	0.5	*0.0002*

Korea		n = 4		n = 3		
	LPS	7.7	11.9	5.7	10.0	
	MuAc	4.8	10.7	3.3	7.5	
	Erg	5.7	9.3	2.2	4.2	*0.005*

Vietnam		n = 2		n = 5		
	LPS	13.4	17.2	7.2	12.9	*0.03*
	MuAc	3.7	6.8	1.4	6.3	
	Erg	6.6	7.3	1.2	2.4	*0.00001*

ns = not studied; LPS = lipopolysaccharides (pmol/mg); MuAc = muramic acid (ng/mg); Erg = ergosterol (ng/mg).

There were no statistically significant differences between the microbial marker contents of "light" and "full flavor" cigarettes of the same international brand purchased in the five different countries (Table 2).

**Table 2:** Microbial components in tobacco from light and full flavor cigarettes of an international brand purchased in five different countries.

	**Full flavor **(n = 5)		**Light **(n = 5)	
		
	mean	SD	mean	SD
**LPS**	12.04	2.57	13.24	2.46
**MuAc**	6.74	0.68	6.50	1.74
**Erg**	9.33	2.35	8.58	1.33

LPS = lipopolysaccharides (pmol/mg); MuAc = muramic acid (ng/mg); Erg = ergosterol (ng/mg).

**Figure 1:**
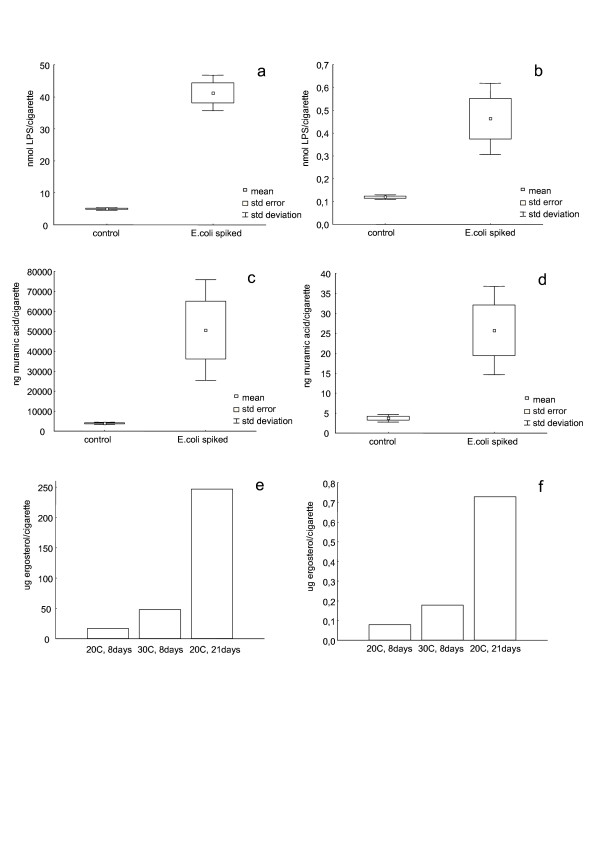
**Microbial components in tobacco (a, c, e) and smoke (b, d, f) of cigarettes i) before and after adding a culture of *E. coli *to the tobacco (a-d), ii) after storing cigarettes at 94% relative humidity under different conditions (e, f)**. LPS = lipopolysaccharide.

Storing the cigarettes at 54 and 75% RH did not affect the tobacco marker composition. By contrast, at 94% RH the MuAc concentration increased 4-fold (20°C, 21 days) and the Erg concentrations increased 3-fold (30°C, 8 days), 18-fold (20°C, 21 days), and 16-fold (30°C, 21 days) (Table 3, Figure 1). Cigarettes stored at 94% RH for 21 days exhibited a greenish colour from clearly visible mold growth.

**Table 3:** Microbial components in tobacco of cigarettes of an international brand stored at different conditions.

		**LPS**	**Erg**	**MuAc**
*RH 54%*				
2 days	20°C	8.3	12.0	4.8
	30°C	8.6	11.9	5.2
8 days	20°C	9.7	13.5	5.8
	30°C	9.3	12.4	3.2
21 days	20°C	8.8	12.3	6.5
	30°C	7.2	14.8	5.0
				
*RH 75%*				
2 days	20°C	9.8	17.2	5.3
	30°C	9.5	13.2	6.4
8 days	20°C	9.8	19.0	5.4
	30°C	8.8	16.0	7.1
21 days	20°C	8.7	14.1	6.8
	30°C	8.1	14.3	4.1
				
*RH 94%*				
2 days	20°C	7.6	15.1	4.4
	30°C	9.8	13.8	4.7
8 days	20°C	9.3	16.8	4.8
	30°C	10.5	48.3	4.6
21 days	20°C	6.2	272	19.7
	30°C	7.3	247	4.6

LPS = lipopolysaccharides (nmol/cigarette); Erg = ergosterol (μg/cigarette); MuAc = muramic acid (μg/cigarette); RH = relative humidity.

### Smoke

Tobacco with added *E. coli *cells contained approximately an 8-fold larger amount of LPS than tobacco without added bacteria (mean values 5.0 respectively 41.2 nmol/cigarette); the increase in the mainstream smoke was approximately 4-fold (0.12 v. 0.46 nmol). For MuAc the corresponding increases were 13-fold (tobacco, mean values 3900 v. 50600 ng) and 7-fold (smoke, mean values 3.72 v. 25.7 ng). The amount of LPS and MuAc in the mainstream smoke from a cigarette was 2.4% and 0.1% respectively (control) and 1.1% and 0.05% respectively (spiked with *E. coli*) of the total amount in the cigarette (Table 4). Mainstream smoke from cigarettes that had been stored for 21 days at 94% RH, containing 15 times more Erg than cigarettes stored at low humidity, contained 9 times increased Erg concentration. The amount of Erg in the mainstream smoke from a cigarette was approximately 0.4% of the total amount in the cigarette (Table 5). See Figure 1.

**Table 4:** Bacterial components in tobacco and smoke collected from cigarettes spiked/not spiked by a suspension of *E. coli*.

	**Tobacco**		**Smoke**	
		
	control	*E. coli *spiked	control	*E. coli *spiked
	4.91	46.7	0.13	0.63
**LPS**	5.45	35.6	0.11	0.44
	4.63	41.4	0.12	0.32
				
	4120	79600	3.94	37.4
**MuAc**	3270	39000	4.54	24.4
	4310	33300	2.69	15.4

LPS = Lipopolysaccharides (nmol/cigarette); MuAc = muramic acid (ng/cigarette).

**Table 5:** Ergosterol in tobacco and smoke from cigarettes after storage at 94% RH.

	**Erg**	
Storage	**Tobacco**	**Smoke**
20°C, 8 days	16.8	0.08
30°C, 8 days	48.3	0.18
30°C, 21 days	247	0.73

Erg = ergosterol (μg/cigarette).

### Tobacco leaves

The concentrations of LPS (5.48–18.8 pmol/mg) and MuAc (1.22 – 4.22 ng/mg) in the studied leaves did not differ from those seen in cigarette tobacco regardless tobacco sort and stage of the curing. A very wide range (0.79 – 74.4 ng/mg) in the concentrations of Erg was found particularly among the F-samples. There was no consistent change of marker concentrations over time (Table 6).

**Table 6:** Microbial components in tobacco leaves collected from a tobacco plantation and a tobacco manufacturing plant.

**Origin of tobacco leaf**	**LPS**	**MuAc**	**Erg**
F-1	6.07	1.68	0.79
F-2	7.97	4.22	74.4
F-3	8.20	2.08	54.4
F-4	9.52	2.45	12.3
V-1	6.44	1.92	2.03
V-2	7.03	2.13	1.73
V-3	7.36	1.73	1.95
B-1	7.66	1.22	52.4
B-2	6.66	1.72	10.9
B-3	8.68	3.40	39.2
K-1	10.97	1.77	16.7
K-2	10.67	2.19	10.2
K-3	7.87	1.67	2.9
SM-1	5.48	1.44	14.5
SM-2	18.8	2.95	35.0
SM-3	7.51	1.71	15.5

LPS = lipopolysaccharide (pmol/mg); MuAc = muramic acid (ng/mg); Erg = ergosterol (ng/mg); F = samples from a tobacco plantation: Fresh leaves (F-1), and leaves after 2 wk (F-2), 6 wk (F-3), and > 6 wk (F-4) of curing; V = Virginia; B = Burley; K = Kentucky; SM = Skroniowski Mocny; 1 = air-dried leaves; 2 = leaves after curing; 3 = leaves after curing and storage > 1 year.

Mesophilic bacteria dominated among the cultured microorganisms and were seen in all studied samples. Gram-negative bacteria and fungi were found in all fresh leaf samples and in approximately half of the cured leaf samples (Tables 7 and 8). *Bacillus *spp. and Gram-positive cocci were seen in most of the cured leaves (Table 9). A range of different bacterial and fungal species were occasionally found (Tables 10, 11 and 12), including *Pantoea agglomerans *(synonyms: *Erwinia herbicola*, *Enterobacter agglomerans*) which was most numerous among Gram-negative bacteria.

**Table 7:** Microorganisms in tobacco leaves (CFU × 10^3^/g) collected from a tobacco plantation.

	**F-1**	**F-2**	**F-3**	**F-4**
Total mesophilic bacteria	2.0	2.4	3.3	6.6
Gram-negative bacteria	0.4	0.5	1.0	2.0
Fungi	0.3	0.3	1.3	2.6

F = samples from a tobacco plantation: Fresh leaves (F-1), and leaves after 2 wk (F-2), 6 wk (F-3), and > 6 wk (F-4) of curing

**Table 8:** Microorganisms in tobacco leaves (CFU × 10^3^/g) collected from a tobacco manufacturing plant.

	**V-1**	**V-2**	**V-3**	**B-1**	**B-2**	**B-3**	**K-1**	**K-2**	**K-3**	**SM-1**	**SM-2**	**SM-3**
Total mesophilic bacteria	381	102.5	8	2	680	155	1	33	11	328	556.5	9
Gram-negative bacteria	145	19.5	0	0	0	38.5	0	0	0	150	415	0
Thermophilic actinomycetes	0.5	0	0	0	0	0	0	0	0	0.5	1.5	2
Fungi	0	7	0.5	1	0	0	6.5	3.5	0	1	8	0

V = Virginia; B = Burley; K = Kentucky; SM = Skroniowski Mocny; 1 = air-dried leaves; 2 = leaves after curing; 3 = leaves after curing and storage > 1 year.

**Table 9:** Mesophilic bacteria in tobacco leaves (CFU × 10^3^/g) collected from a tobacco manufacturing plant.

	**V-1**	**V-2**	**V-3**	**B-1**	**B-2**	**B-3**	**K-1**	**K-2**	**K-3**	**SM-1**	**SM-2**	**SM-3**
Gram-negative bacteria	325	20	0	0	0	17	0	0	0	200	510	0
Gram-positive cocci	5	2.5	1.5	1.5	0	0	0.5	0	1	10	10	0
Endospore-forming bacilli	15^b^	75.5^a^	6.5^b^	0.5^b^	680^a^	111^a,b^	0.5^b^	33^b^	10^b^	6.5^b^	30^b^	9^b^
Coryneform bacteria	35^c,d^	0	0	0	0	27^e^	0	0	0	108^d,e^	1^f^	0
Mesophilic actinomycetes	1^g^	4.5^g^	0	0	0	0	0	0	0	3.5^g,h^	5.5^g,h^	0

**Total**	**381**	**102.5**	**8**	**2**	**680**	**155**	**1**	**33**	**11**	**328**	**556.5**	**9**

V = Virginia; B = Burley; K = Kentucky; SM = Skroniowski Mocny; 1 = air-dried leaves; 2 = leaves after curing; 3 = leaves after curing and storage > 1 year.

^a^*Bacillus subtilis*, ^b^*Bacillus *spp., ^c^*Arthrobacter ilicis*, ^d^*Curtobacterium pusillum*, ^e^*Sanguibacter inulinus*, ^f^*Arcanobacterium pyogenes*, ^g^*Streptomyces albus*, ^h^*Streptomyces *spp.

**Table 10:** Gram-negative bacteria in tobacco leaves (CFU × 10^3^/g) collected from a tobacco manufacturing plant.

	**V-1**	**V-2**	**V-3**	**B-1**	**B-2**	**B-3**	**K-1**	**K-2**	**K-3**	**SM-1**	**SM-2**	**SM-3**
*Pantoea agglomerans*	145	3	0	0	0	6	0	0	0	90	405	0
Other Enterobacteriaceae species	0	16.5^b,c^	0	0	0	15.5^a^	0	0	0	0	0	0
*Acinetobacter calcoaceticus*	0	0	0	0	0	9	0	0	0	0	0	0
Pseudomonadaceae species	0	0	0	0	0	8^e,f^	0	0	0	60^f,g^	10^d^	0

**Total**	145	19.5	0	0	0	38.5	0	0	0	150	415	0

V = Virginia; B = Burley; K = Kentucky; SM = Skroniowski Mocny; 1 = air-dried leaves; 2 = leaves after curing; 3 = leaves after curing and storage > 1 year.

^a^*Buttiauxella brennerae*, ^b^*Enterobacter amnigenus*, ^c^*Enterobacter cancerogenus*, ^d^*Pseudomonas fluorescens*, ^e^*Pseudomonas viridilivida*, ^f^*Sphingomonas paucimobilis*, ^g^*Stenotrophomonas maltophilia*.

**Table 11:** Thermophilic actinomycetes in tobacco leaves (CFU × 10^3^/g) collected from a tobacco manufacturing plant.

	**V-1**	**V-2**	**V-3**	**B-1**	**B-2**	**B-3**	**K-1**	**K-2**	**K-3**	**SM-1**	**SM-2**	**SM-3**
*Thermoactinomyces thalpophilus*	0	0	0	0	0	0	0	0	0	0.5	0	2
*Thermoactinomyces vulgaris*	0.5	0	0	0	0	0	0	0	0	0	1.5	0

**Total**	0.5	0	0	0	0	0	0	0	0	0.5	1.5	2

V = Virginia; B = Burley; K = Kentucky; SM = Skroniowski Mocny; 1 = air-dried leaves; 2 = leaves after curing; 3 = leaves after curing and storage > 1 year.

**Table 12:** Fungi in tobacco leaves (CFU × 10^3^/g) collected from a tobacco manufacturing plant.

	**V-1**	**V-2**	**V-3**	**B-1**	**B-2**	**B-3**	**K-1**	**K-2**	**K-3**	**SM-1**	**SM-2**	**SM-3**
*Aspergillus *spp.	0	5.5^a,b,c^	0	0	0	0	4^d^	0	0	1^a,b^	0	0
Other fungi	0	1.5^f,j^	0.5^g^	1^h^	0	0	2.5^e,l^	3.5^k^	0	0	8^i^	0

**Total**	**0**	**7**	**0.5**	**1**	**0**	**0**	**6.5**	**3.5**	**0**	**1**	**8**	**0**

V = Virginia; B = Burley; K = Kentucky; SM = Skroniowski Mocny; 1 = air-dried leaves; 2 = leaves after curing; 3 = leaves after curing and storage > 1 year.

^a^*Aspergillus fumigatus*, ^b^*Aspergillus glaucus*, ^c^*Aspergillus niger. *^d^*Aspergillus penicilloides*, ^e^*Alternaria alternata*, ^f^*Cladosporium herbarum*, ^g^*Oidiodendron citrinum*, ^h^*Penicillium brevi-compactum*, ^i^*Penicillium *spp., ^j^*Rhizopus nigricans*, ^k^*Rhodotorula rubra*, ^l^Yeasts (white colonies).

## Discussion

Tobacco is an agricultural product rich in microorganisms that naturally colonize the tobacco plants [10]. We found that mesophilic bacteria dominated among the bacteria in both fresh and cured tobacco leaves but also that a wide range of other bacteria and fungi were present too. It is noteworthy that among Gram-negative bacteria recovered from tobacco leaves prevailed the species *Pantoea agglomerans *possessing strong endotoxic and allergenic properties [9]. This species represents probably one of the most important sources of endotoxin in tobacco. Interestingly, the bacterial biomass (MuAc) and LPS (3-OH FAs) concentrations in fresh tobacco leaves were in the same range as in the cured leaves and in the final tobacco product demonstrating that there is little enrichment of bacteria after the leaves are being collected at the plantation. By contrast, the concentrations of Erg varied dramatically especially between V- and B-samples, and between the different F-samples. The concentrations of the studied microbial components in the smoke were in general agreement with the concentrations in cigarette tobacco. Thus our results demonstrate that the microbiological material that is present in tobacco smoke originates from microorganisms that colonize the tobacco plants in the fields.

The microbial (marker) concentrations were much lower in some cigarettes locally produced in China, Korea, and Vietnam than in cigarettes of international brands purchased in the same countries. The reason for this finding is unknown; however, pesticides and fungicides – which would reduce microbial growth on the plants – are common on tobacco plantations. Indeed, pesticides have been identified in cigarette tobacco [11]. We found that storing cigarettes at 94% RH for 8 days or more may result in bacterial and, more pronounced, fungal growth in the cigarette tobacco; this may lead to increased concentrations of microbiological agents in the smoke. No differences in microbial load in "light" and "full flavor" cigarettes were found.

The endotoxin that is present in tobacco smoke may be responsible for some of the health effects of smoke. For example, it has been shown that asthmatics' symptoms are worsened in indoor environments that contain relatively larger concentrations of endotoxins [12]; interestingly, such symptoms are typically worsened also by exposure to tobacco smoke. The present study demonstrates that tobacco smoke contains bacterial components other than endotoxin as well as fungal components; indeed, aflatoxin B1 has been demonstrated in sidestream smoke [13]. Bacterial peptidoglycan, sometimes called the "endotoxin of Gram-positive bacteria"[14], is a potent entity that use Toll-like receptor (TLR)-2 for cell binding and activation of the innate immunity response (endotoxin uses TLR-4); inhalation of dust containing elevated concentrations of peptidoglycan or fragments thereof has been shown to result in fever and increased levels of blood interleukin-6 [15].

In conclusion, tobacco smoke is a bioaerosol that contains endotoxin, peptidoglycan or peptidoglycan fragments, and various fungal constituents. This knowledge may help to explain the high prevalence of respiratory disorders such as bronchoalveolar neutrophilia, airway obstruction, and bronchial hyperresponsiveness among smokers and individuals exposed to second hand smoke, since these symptoms are also commonly associated with exposure to bioaerosols [16-19]. Public awareness that tobacco smoke contains high concentrations of bacterial and fungal constituents may contribute to reduce smoking.

## Authors' contributions

LL designed the study and did the main writing. BS and JD were responsible for the samplings and microbiological analyses. CP and BR made the chemical analyses and EK and JS made the culturing and microbiological analyses. All authors participated in the writing.
